# HMGA1 positively regulates the microtubule-destabilizing protein stathmin promoting motility in TNBC cells and decreasing tumour sensitivity to paclitaxel

**DOI:** 10.1038/s41419-022-04843-4

**Published:** 2022-05-03

**Authors:** Michela Sgubin, Silvia Pegoraro, Ilenia Pellarin, Gloria Ros, Riccardo Sgarra, Silvano Piazza, Gustavo Baldassarre, Barbara Belletti, Guidalberto Manfioletti

**Affiliations:** 1 Department of Life Sciences, University of Trieste, Trieste, Italy; 2 Division of Molecular Oncology, Centro di Riferimento Oncologico di Aviano (CRO), IRCCS, National Cancer Institute, Aviano, Italy; 3 International Centre for Genetic Engineering and Biotechnology (ICGEB), Padriciano 99, Trieste, Italy; 4 Present Address: International School for Advanced Studies (SISSA), Area of Neuroscience Trieste, Trieste, Italy

**Keywords:** Breast cancer, Breast cancer

## Abstract

High Mobility Group A1 (HMGA1) is an architectural chromatin factor involved in the regulation of gene expression and a master regulator in Triple Negative Breast Cancer (TNBC). In TNBC, HMGA1 is overexpressed and coordinates a gene network that controls cellular processes involved in tumour development, progression, and metastasis formation. Here, we find that the expression of HMGA1 and of the microtubule-destabilizing protein stathmin correlates in breast cancer (BC) patients. We demonstrate that HMGA1 depletion leads to a downregulation of stathmin expression and activity on microtubules resulting in decreased TNBC cell motility. We show that this pathway is mediated by the cyclin-dependent kinase inhibitor p27^kip1^ (p27). Indeed, the silencing of HMGA1 expression in TNBC cells results both in an increased p27 protein stability and p27-stathmin binding. When the expression of both HMGA1 and p27 is silenced, we observe a significant rescue in cell motility. These data, obtained in cellular models, were validated in BC patients. In fact, we find that patients with high levels of both HMGA1 and stathmin and low levels of p27 have a statistically significant lower survival probability in terms of relapse-free survival (RFS) and distant metastasis-free survival (DMFS) with respect to the patient group with low HMGA1, low stathmin, and high p27 expression levels. Finally, we show in an in vivo xenograft model that depletion of HMGA1 chemo-sensitizes tumour cells to paclitaxel, a drug that is commonly used in TNBC treatments. This study unveils a new interaction among HMGA1, p27, and stathmin that is critical in BC cell migration. Moreover, our data suggest that taxol-based treatments may be more effective in reducing the tumour burden when tumour cells express low levels of HMGA1.

## Introduction

HMGA1, a member of the High Mobility Group A (HMGA) family, is an architectural transcription factor able to modulate chromatin structure and regulate gene expression [1–3]. Even though HMGA1 has no transcriptional activity per se, it contributes to gene expression regulation by altering general chromatin status through the competition with histone H1 and by binding to DNA and/or transcription factors leading to the formation of stereospecific macromolecular complexes called “enhanceosomes”, crucial for transcription activation [4, 5]. HMGA1 is a highly connected nuclear factor [5–7] and for this reason, it is considered a central hub in the chromatin network and a “master” gene whose alteration of expression can affect the regulation of a vast set of genes with a large impact on cell phenotype [8, 9].

HMGA1 is defined as an oncofetal protein since it is highly expressed during embryogenesis, its expression decreases to very low levels or it is absent in adults but then it is overexpressed in a variety of tumours [10]. Indeed, several works demonstrated that HMGA1 expression is correlated with high tumour grade and metastasis formation, resistance to therapies and poor prognosis in a large set of human malignant neoplasias, and it is directly involved in the development and progression of cancer [1, 11–16].

HMGA1 has been investigated in breast cancer (BC) and in particular in the triple-negative subtype (TNBC) [1, 11, 17]. A causal role of HMGA1 in BC onset and development has been demonstrated. Indeed, HMGA1 overexpression in non-tumorigenic human breast epithelial cells leads to the acquisition of a transformed and aggressive phenotype [18], whereas silencing of HMGA1 expression in highly aggressive TNBC cell lines causes a reversion of the tumorigenic phenotype in vitro and in vivo [19, 20].

Transcriptomic approaches have demonstrated that HMGA1 controls a gene network involved in critical processes in BC such as epithelial-to-mesenchymal transition (EMT), stemness, cell proliferation, migration, and invasion [19–22]. In addition, data suggest that HMGA1 might promote chromatin relaxation through a histone H1-mediated mechanism, impacting nuclear stiffness and thus favouring the invasiveness of cancer cells [23]. Recently, a bioinformatics approach further pointed out HMGA1 as a key gene in TNBC underlying its relevance as a hub in controlling gene networks [8].

Stathmin (also known as oncoprotein 18/Op18) is a microtubule-destabilizing phosphoprotein often overexpressed in metastatic tumours [24]. Stathmin microtubule-destabilizer activity is exerted either by directly promoting the microtubule catastrophe or by sequestering the free αβ-tubulin heterodimers in a stable tubulin/stathmin (T_2_S) ternary complex, thus preventing tubulin incorporation in growing microtubules [24]. Given its activity as a microtubule-destabilizer, stathmin is involved in the regulation of the cell cycle, in fact alterations in its expression levels are associated with severe mitotic spindle abnormalities, altered M phase progression, and apoptosis [25]. Stathmin expression and activity are important in the control of cell morphology and motility in vitro [26] and to maintain oriented cell division and apicobasal polarity in normal mammary glands in vivo [27]. Stathmin is also critical to establish a pro-tumorigenic program that eventually sustains HER2-positive BC formation in mice.

Stathmin is ubiquitously expressed in human and mouse tissues with a higher expression detected in the nervous system and in embryonic with respect to adult tissues. With the exception of neuronal cells, in general, stathmin expression is induced by pro-mitogenic stimuli and restricted to the proliferating compartment in almost all tissues [28, 29]. Stathmin expression and/or activity is upregulated in nearly all types of human cancer, including BC, and it is almost invariably associated with increased local invasion and metastasis formation suggesting that it could play a major role in both tumour onset and progression [24, 27]. Finally, it is relevant the protective activity of stathmin toward microtubule-targeting drugs, such as vinca alkaloids and taxanes [24].

It has been demonstrated that stathmin activity may be modulated by the interaction with the cell cycle inhibitor p27 [30–34]. In this paper we demonstrate the existence of an HMGA1/p27/stathmin axis that regulates the motility of TNBC cells. The silencing of HMGA1 expression in TNBC cells leads to a decrease in stathmin expression levels and activity on microtubules, impairing cell motility in a p27-dependent manner. Moreover, we show in a xenograft mouse model that HMGA1-depleted TNBC cells are more sensitive to paclitaxel. These findings suggest that taxol-based treatments may be more efficacious in patients expressing low levels of HMGA1.

## Results

### Stathmin expression correlates with HMGA1 expression in breast cancer

**Fig. 1:**
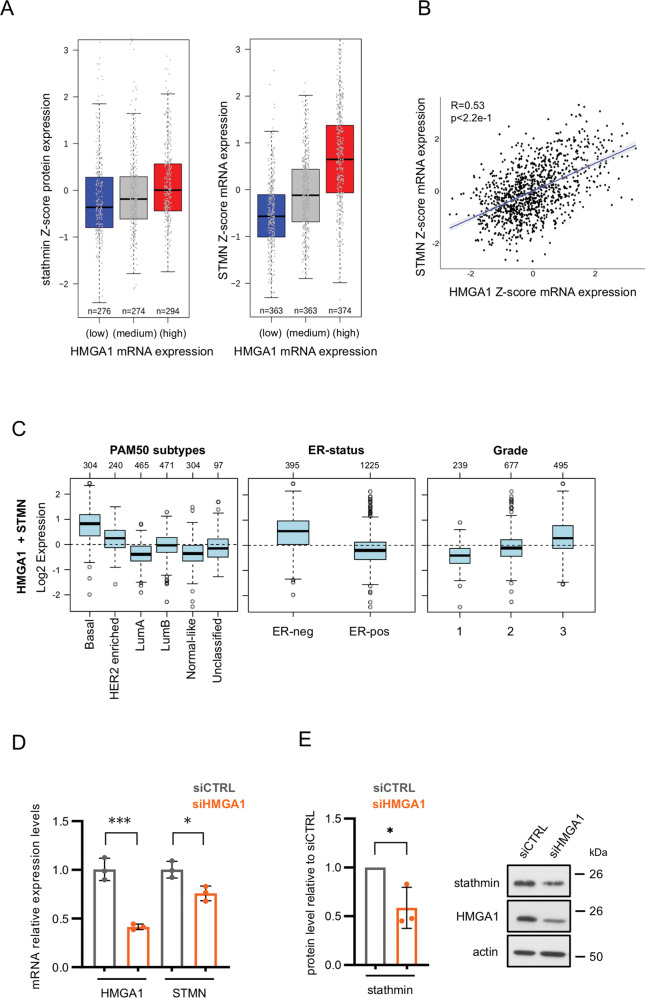
Stathmin expression correlates with HMGA1 in breast cancer. **A** Boxplot analysis showing the correlation between the mRNA expression of HMGA1 (low/medium/high) with stathmin protein (left) and mRNA (right) expression in a cohort of 844 TCGA breast cancer patients. **B** Scatter plots of gene expression data from the TCGA BC dataset using the Z-score normalized counts for the genes of interest. The regression line is plotted and the grey bands around the line represent the standard error. R is the correlation between the two genes with the corresponding *p*-value test. **C** Co-expression of HMGA1 and STMN among BC subtypes, ER-status, and tumour grade. The analyses were performed investigating the Gene expression-based Outcome for Breast cancer Online (GOBO) tool. **D** STMN mRNA expression analysis with qRT-PCR in MDA-MB-231 cells after 72 h of HMGA1 silencing. GAPDH was used for normalization. The data are compared to control condition and are presented as the mean ± SD (*n* = 3). *P < 0.05, ***P < 0.001; two-tailed Student’s *t*-test. **E** The graph represents western blot densitometry analysis of stathmin protein levels in MDA-MB-231 cells after 72 h of HMGA1 silencing. The data are compared to control condition and are presented as mean ± SD (*n* = 3). Actin was used to normalize for protein expression. *P < 0.05; two-tailed Student’s *t*-test. Representative western blot is shown on the right.

To characterize the molecular networks controlled by HMGA1 in sustaining TNBC aggressiveness, we analyzed the TCGA protein expression database of BC patients, searching for alterations in the expression level of proteins in patients with alterations in HMGA1 mRNA expression. In particular, we stratified patients according to their HMGA1 expression and ranked the differential changes obtained from the expression of other proteins (Supplementary Tables 1 and 2). From the results obtained we focused on those with a clear role in tumour aggressiveness. Among the most significant outputs, we found a direct relation between HMGA1 and stathmin protein and mRNA expression (Fig. 1A, B). To further examine this relationship, we analyzed the expression of both HMGA1 and stathmin in 1881 BC samples, thanks to the Gene expression-based Outcome for Breast Cancer Online (GOBO) tool, in which all clinical information of the patients is available. Among BC patients both HMGA1 and stathmin are expressed at high levels in the basal-like and the HER2+ subtypes, ER-negative, and high-grade tumours (Fig. 1C and Supplementary Fig. 1). Therefore, in accordance with the co-expression observed between the two factors, HMGA1 and stathmin are enriched in BC patients with the most aggressive features.

Based on these results, we then asked if HMGA1 regulates stathmin expression in TNBC cell lines. To this aim, we silenced HMGA1 expression in MDA-MB-231 cells and looked at stathmin mRNA and protein expression levels. HMGA1 silencing induced a downregulation of stathmin, both at the mRNA and at the protein levels (Fig. 1D, E). These results were confirmed in the other two TNBC cell lines, MDA-MB-157 and MDA-MB-468, and with a different siRNA against HMGA1 (Supplementary Fig. 2).

### HMGA1 regulates cell migration through stathmin-mediated microtubules modulation

**Fig. 2:**
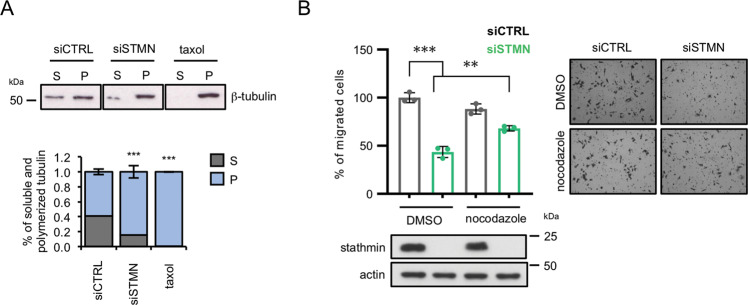
Stathmin regulates cell migration through microtubule modulation. **A** Western blot analysis of tubulin protein levels in soluble-tubulin (S) and polymerized-tubulin (P) fractions after microtubule separation assay performed in MDA-MB-231 cells silenced for stathmin expression (siSTMN), control (siCTRL), or taxol (a drug that stabilizes microtubules counteracting their disassembling) as positive control of tubulin polymerization. Densitometry analysis of tubulin expression (normalized on total tubulin amount of each sample) is reported in the bottom graphs and presented as the mean (±SD) of *n* = 3 replicates. **B** Quantification of the trans-well migration assay performed in MDA-MB-231 cells upon silencing of stathmin (siSTMN) and 1 h of nocodazole treatment. The results are presented as the mean of the percentage of migrated cells with respect to control (*n* = 3). Representative pictures are shown on the right of the graph. Stathmin western blot analyses are reported at the bottom.

Stathmin is a microtubule-destabilizer that can modulate cell migration through the regulation of the microtubule dynamics [24, 26]. Therefore, we firstly investigated whether stathmin is involved in promoting the destabilization of the microtubule cytoskeleton of MDA-MB-231 cells. For this purpose, we silenced stathmin and analyzed the amount of the polymerized and the soluble-tubulin fractions. This analysis showed an increase in the polymerized tubulin over the free one in cells silenced for stathmin (Fig. 2A and Supplementary Fig. 3), confirming that also in MDA-MB-231 cells stathmin actively promotes microtubule dynamics.

**Fig. 3:**
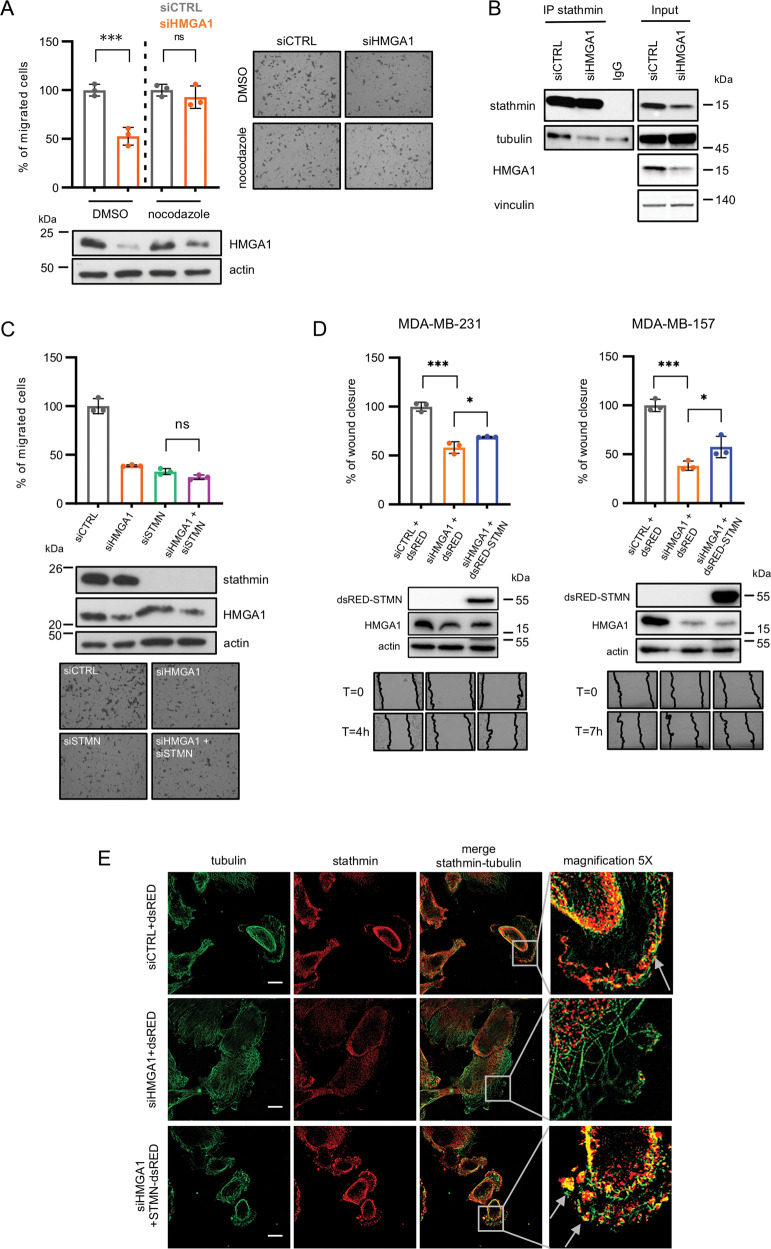
HMGA1 regulates cell migration through stathmin activity. **A** Quantification of the trans-well migration assay performed in MDA-MB-231 cells upon silencing of HMGA1 and 1 h of nocodazole treatment. The results are presented as the mean of the percentage of migrated cells with respect to control (*n* = 3). Representative pictures are reported on the right of the graph. HMGA1 western blot analyses are reported at the bottom. **B** Lysates from MDA-MB-231 cells transfected with siCTRL and siHMGA1 were immunoprecipitated with stathmin and non-specific IgG antibodies. The amount of co-immunoprecipitated tubulin is visualized by western blot analysis. Inputs are shown on the right. **C** Quantification of the trans-well migration assay performed in MDA-MB-231 cells upon silencing of HMGA1 (siHMGA1) and stathmin (siSTMN). The results are presented as the mean of the percentage of migrated cells with respect to control (*n* = 3). HMGA1 and stathmin western blot analysis and representative pictures are reported at the bottom. **D** Quantification of wound-healing assays performed in MDA-MB-231 (left) and MDA-MB-157 (right) cells upon silencing of HMGA1 (siHMGA1) and transfection with the stathmin expression plasmid (dsRED-STMN). The results are presented as the mean of wound closure with respect to control (*n* = 3). Representative pictures of HMGA1 and dsRED-STMN western blot analysis and wound healing are shown at the bottom. **E** Representative immunofluorescence analysis of stathmin (red) and tubulin (green) at the migratory front of MDA-MB-157 cells. The cells were silenced or not for HMGA1 and transfected with the stathmin expression plasmid (dsRED-STMN). Arrows point the edges where both stathmin and tubulin are present. Images were taken with lattice SIM at ×60 magnification. Scale bar, 10 µm.

To investigate whether stathmin is involved in cell migration through the depolymerization of microtubules, we performed a trans-well migration assay in MDA-MB-231 cells depleted for stathmin and treated, or not, with nocodazole, a microtubule depolymerizing drug. This drug was used to prevent microtubule polymerization induced by stathmin silencing. Stathmin depletion induced a strong reduction of cell migration abilities that was recovered by nocodazole treatment (Fig. 2B), indicating that stathmin modulates cell migration through the regulation of microtubules polymerization. Intriguingly, we observed that nocodazole treatment was able to rescue the reduced cell migration induced by HMGA1 silencing as well (Fig. 3A), indicating that, in analogy to what observed for stathmin, microtubule dynamics are an essential component in the HMGA1 regulation of cell migration. To explore a possible connection between HMGA1 and stathmin-mediated tubulin modulation, we performed Co-IP experiments demonstrating that in HMGA1 silenced MDA-MB-231 cells the interaction between stathmin and tubulin was reduced with respect to control cells (Fig. 3B). We then analyzed cell migration through trans-well assay in MDA-MB-231 cells depleted for both HMGA1 and stathmin. The co-silencing of HMGA1 and stathmin promoted a reduction in cell migration comparable to that induced by HMGA1 silencing (Fig. 3C), suggesting that stathmin operates downstream HMGA1 in cell migration control. To finally verify if stathmin is a downstream effector of HMGA1, we overexpressed stathmin in HMGA1-depleted MDA-MB-231 and MDA-MB-157 cells showing a rescue in cell migration (Fig. 3D).

In accord with the above-collected data, using high-resolution immunofluorescence analysis, we observed that in control cells both stathmin and tubulin localized at the edges of migrating cells while in HMGA1 silenced cells this co-localization is lost (Fig. 3E and Supplementary Fig. 4) but it is recovered following stathmin overexpression (Fig. 3E).

Altogether, these data indicate that HMGA1 regulates migration through stathmin-mediated microtubules modulation.

### HMGA1 regulates the cyclin-dependent kinase inhibitor p27

**Fig. 4:**
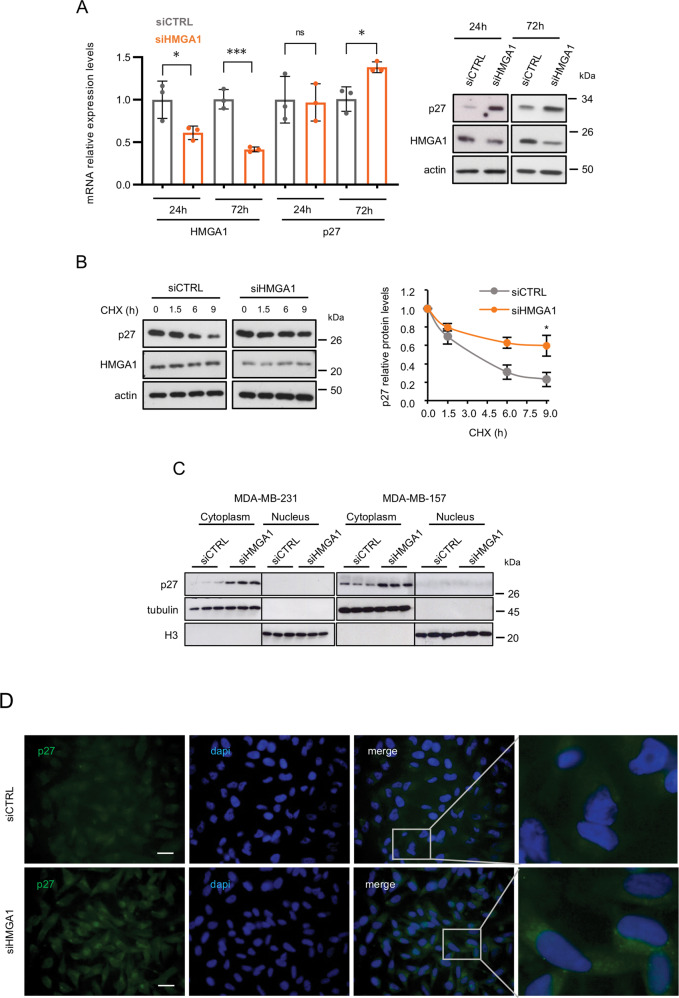
HMGA1 regulates p27 protein expression. **A** p27 mRNA expression analysis with qRT-PCR in MDA-MB-231 cells after 24 and 72 h of HMGA1 silencing. GAPDH was used for normalization. The data are compared to control condition and are presented as the mean ± SD (*n* = 3). *P < 0.05, ***P < 0.001; two-tailed Student’s *t*-test (left). Representative western blot of p27 in MDA-MB-231 cells upon 24 and 72 h of HMGA1 silencing. Actin was used as a loading control (*n* = 3) (right). **B** Representative western blot analysis of p27 expression in MDA-MB-231 cells silenced or not for HMGA1 (48 h) and treated with cycloheximide (CHX) for 0, 1.5, 6, and 9 h. Densitometry analyses of p27 expression (normalized with respect to actin expression) are reported on the right. Data are presented as mean ± SEM (*n* = 3). *P < 0.05; two-way ANOVA test. **C** Western blot analysis of p27 expression in cytoplasmic and nuclear fractions of MDA-MB-231 and MDA-MB-157 after 72 h of HMGA1 silencing. Tubulin was used as cytoplasmic control protein and histone H3 as nuclear control protein. **D** Representative immunofluorescence analysis of p27 localization in MDA-MB-231 cells after 72 h of HMGA1 silencing. On the right, magnification of the merged staining. Images were taken at ×60 magnification. Scale bar, 20 µm.

The cyclin-dependent kinase inhibitor p27 is one of the cytoplasmic regulators of stathmin activity. In particular, the expression of p27 affects microtubule stability due to its ability to bind and impair the function of stathmin, therefore influencing cancer cell migration and invasion [30]. We thus explored the possibility that p27 could mediate the effect observed by silencing HMGA1 on stathmin activity. Intriguingly, the expression of p27 protein and mRNA inversely correlates with the expression of HMGA1 in the TCGA protein expression database of BC patients (Supplementary Fig. 5). In addition, the expression of p27 mRNA in BC patients inversely correlates with that of HMGA1, being lower in the basal-like and HER2+ subtypes, with respect to the other subtypes, in the ER-negative subgroup and in high-grade tumours (Supplementary Fig. 1). Moreover, when we silenced HMGA1 expression in MDA-MB-231 cells we observed a quick upregulation of p27 protein levels (Fig. 4A) that was confirmed in other TNBC cell lines as well (Supplementary Fig. 6) while the p27 mRNA was weakly upregulated and only at later time points (Fig. 4A).

To further investigate the mechanism of p27 upregulation by HMGA1, we analyzed the effect of HMGA1 on p27 protein stability by cycloheximide. MDA-MB-231 cells were silenced for HMGA1 expression and then protein synthesis was blocked by the addition of cycloheximide and the relative p27 protein levels were analyzed at different time points (1.5, 6, and 9 h) by immunoblotting, and protein abundance was quantified by densitometry analysis (Fig. 4B). The presence of HMGA1 had an impact on p27 degradation, as demonstrated by the p27 protein half-life, that in control cells was about 3.5 h while in HMGA1-depleted cells of nearly 9 h (Fig. 4B).

It has been reported that cytoplasmic p27 could bind stathmin, affecting microtubules stability [30]. We thus analyzed the localization of p27 in MDA-MB-231 and MDA-MB-157 cells following HMGA1 silencing. Nuclear and cytoplasmic protein fractionation and immunofluorescence analysis showed that p27 was mainly localized in the cytoplasm in control cells and that this localization is maintained following HMGA1 depletion (Fig. 4C, D and Supplementary Fig. 7). Overall, these data indicate that HMGA1 regulates p27 expression affecting its degradation and suggest that the increased cytoplasmic p27 may cause a stathmin-mediated effect on cell migration, observed in HMGA1-depleted TNBC cells.

### HMGA1/p27/stathmin axis promotes migration of MDA-MB-231 cells

**Fig. 5:**
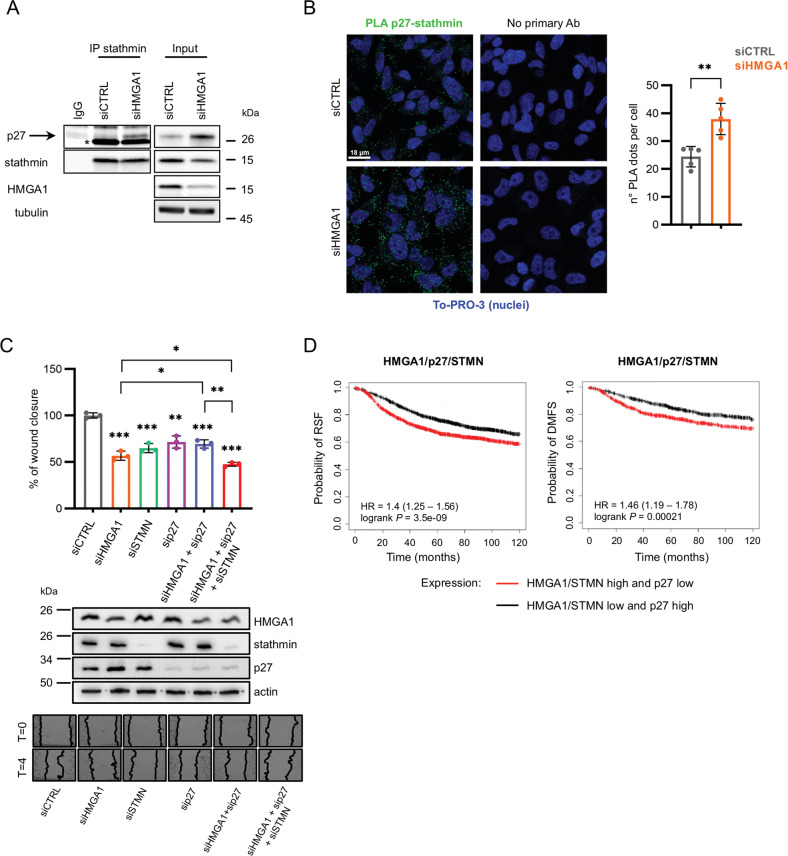
HMGA1/p27/stathmin axis regulates migration in MDA-MB-231 cells. **A** Lysates from MDA-MB-231 cells transfected with siCTRL and siHMGA1 were immunoprecipitated with stathmin and non-specific IgG antibodies. The amount of co-immunoprecipitated p27 is visualized by western blot analysis (the specific band is indicated by the arrow). The asterisk indicates the aspecific band due to the presence of immunoglobulines. Inputs are shown on the right. **B** p27-stathmin proximity was measured by in situ PLA in MDA-MB-231 cells transfected with siCTRL and siHMGA1. In situ PLA is indicated by green signals of the rolling cycle amplification products. Nuclei (blue) were counterstained using TO-PRO-3. Scale bar, 18 μM. Quantification of the number of fluorescent puncta per cell is shown on the right (*n* = 5 different fields). **P < 0.01; two-tailed Student’s *t*-test. **C** Quantification of wound-healing assay performed in MDA-MB-231 cells upon silencing of HMGA1 (siHMGA1), stathmin (siSTMN) and p27 (sip27). The results are presented as the mean of percentage of the wound closure relative to control (*n* = 3). *P < 0.05, **P < 0.01, ***P < 0.001; two-tailed Student’s *t*-test. A representative western blot of HMGA1, stathmin and p27 expression after silencing is reported at the bottom of the graph. Representative pictures are reported on the bottom of the panel. **D** Kaplan–Meier survival curves of Relapse Free Survival (RFS) of 3951 breast cancer patients and Distant Metastasis Free Survival (DMFS) of 1746 BC patients investigated with Kaplan–Meier plotter (KM-plotter, https://kmplot.com/analysis/index.php?p=background) and containing information from GEO, EGA, and TCGA. The data investigated were based on the RNA expression of HMGA1/p27/STMN together (p27 is analyzed in inverted expression).

On the basis of the results above described, we hypothesized the presence of an HMGA1/p27/stathmin axis regulating the motility of TNBC cells, in which, after HMGA1 silencing, the increased amount of p27 protein could bind stathmin, inhibiting its activity on microtubules and, consequently, cell migration. We therefore performed co-immunoprecipitation of stathmin and p27 in HMGA1 silenced MDA-MD-231 cells. Figure 5A shows that, following HMGA1 depletion, there is an enhanced interaction between p27 and stathmin. This result was confirmed with a proximity-ligation-assay (PLA) between p27 and stathmin; in fact, we observed an increase in the number of dots in HMGA1-depleted cells with respect to the control ones (Fig. 5B). We therefore tested if the ability of HMGA1 to promote MDA-MB-231 cell migration was due to the sequestration of stathmin mediated by p27. Using a scratch assay, we observed a decrease in cell migration with the silencing of each of the three factors separately but, notably, we found a significant rescue in cell motility when the expression of p27 was silenced in HMGA1-depleted cells (Fig. 5C). To demonstrate if this rescue was mediated by stathmin activity we silenced the expression of HMGA1, p27, and stathmin together and we observed that stathmin depletion reduced back the cell migration rescued by p27 silencing (Fig. 5C), suggesting that stathmin is downstream to p27 in the HMGA1-mediated regulation of cell migration.

To explore whether these results, obtained in a TNBC cellular model, could be validated in data obtained from patients, we analyzed the clinical relevance of the expression of the three genes in a dataset of BC patients from GEO, EGA, and TCGA. In particular, we investigated the dataset considering p27 mRNA expression inversely correlated with that of HMGA1 and stathmin. Considering together the expression of the three genes, we observed that patients with high levels of HMGA1 and stathmin and low levels of p27 displayed a statistically significant lower survival probability, in terms of relapse-free survival (RFS) and distant metastasis-free survival (DMFS), with respect to the patient group with HMGA1, stathmin low and p27 high (Fig. 5D). Interestingly, in DMFS, the three genes signature has a stronger prognostic value than each of the single genes (Fig. 5D and Supplementary Fig. 8). Overall, clinical datasets analysis confirmed that the relative expression of the three genes has an impact on BC progression, since a signature combining HMGA1, p27, and stathmin expression has a clinical prognostic value. These results support the existence of an HMGA1/p27/stathmin axis playing an important role in the regulation of cell motility and breast tumour progression.

### Depletion of HMGA1 enhances sensitivity to paclitaxel in a xenograft mouse model

**Fig. 6:**
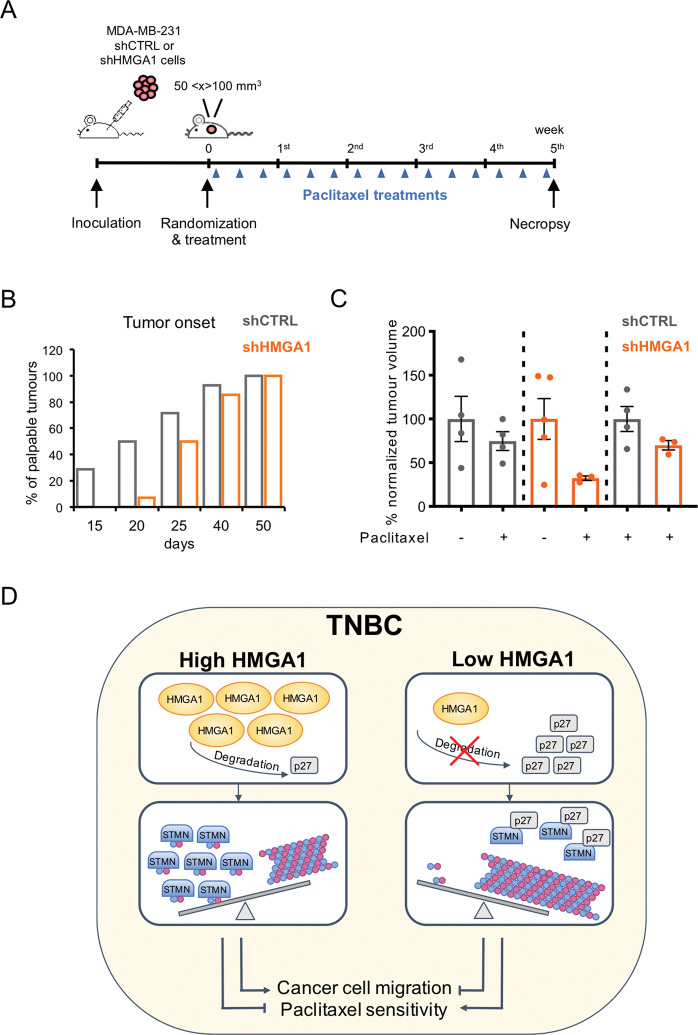
Depletion of HMGA1 enhances sensitivity to paclitaxel in a xenograft mouse model. **A** Flow chart of the experimental BC mouse model for the treatment with paclitaxel. Mice were inoculated with MDA-MB-231/TetR shCTRL or shHMGA1 inducible clones and, when the tumour masses reached 50–100 mm^3^ of tumour volume, they were randomly grouped and then treated with paclitaxel. **B** Evaluation of tumour onset. The graph shows the different timing of tumour onset (tumour mass of 50–100 mm^3^) in MDA-MB-231 shCTRL and MDA-MB-231 shHMGA1 injected cells (*n* = 14 tumours). **C** Graph representing the percentage of volume masses at the endpoint of the treatment, comparing shCTRL (*n* = 4) vs. shCTRL + paclitaxel (*n* = 4), shHMGA1 (*n* = 5) vs. shHMGA1 + paclitaxel (*n* = 3), and shCTRL + paclitaxel vs. shHMGA1 + paclitaxel. Tumour data have been normalized on the first day of the treatment. Data are presented as mean ± SEM. **D** Scheme illustrating the regulation of HMGA1 on p27 and stathmin and its effect on cancer cell migration and paclitaxel sensitivity (blue and pink dots represent free and polymerized tubulin.

The use of standard chemotherapeutics, such as paclitaxel or doxorubicin, is the mainstay of TNBC treatment, with the uprising resistance as a major drawback. Evidence exists that high levels of stathmin may promote paclitaxel resistance in BC [35–38]. By binding to microtubules, paclitaxel stabilizes and protects them from disassembly, which is the opposite function played by the microtubule-destabilizing protein stathmin. Results reported so far demonstrate that silencing the expression of HMGA1 reduces the level and the activity of stathmin on microtubules. Therefore, we asked whether depletion of HMGA1 in a xenograft mouse model could sensitize BC cells to paclitaxel. Control (shCTRL) and HMGA1-depleted (shHMGA1) MDA-MB-231 cells were injected subcutaneously into the fat pads of nude mice and, when tumours reached palpable masses (50–100 mm^3^), mice were treated with paclitaxel three times a week for 5 weeks (Fig. 6A). Tumours generated by shHMGA1 MDA-MB-231 cells started to generate palpable masses later than those from shCTRL MDA-MB-231 cells (Fig. 6B). To assess the efficacy of the combination of paclitaxel treatment with HMGA1 depletion on mouse tumour burden, we measured the volume of the tumour at the endpoint of the treatment when mice were sacrificed. We found that paclitaxel treatment reduced the tumour volume in mice injected with shCTRL MDA-MB-231 cells and with shHMGA1 MDA-MB-231 cells, but this effect is much stronger in HMGA1-depleted cells compared to the control (Fig. 6C). This result indicates that HMGA1 depletion was able to sensitize tumours to the treatment and suggests that taxol-based treatments may be more efficacious in reducing the tumour burden when tumour cells expressed low levels of HMGA1.

## Discussion

The main reason for the poor prognosis of TNBC is the lack of an effective targeted therapy in the early stages, which makes TNBC prone to metastatic relapse [39–42]. Indeed, at a certain point of cancer development, cancer cells increase their motility and acquire the ability to migrate and invade the surrounding tissue. These changes, known as EMT, are also associated with an increase in stem cell properties, resistance to apoptosis, and drug resistance [43].

HMGA1 is a chromatin architectural factor that promotes the metastatic process in TNBC cells by regulating EMT and stemness via the activation of a specific gene signature linked to the Wnt/beta-catenin, Notch, Pin1/mutantp53 and Hippo signalling pathways [19–21].

Given the crucial role of HMGA1 in conferring TNBC aggressiveness through the modulation of specific gene networks and pathways, we dissected other HMGA1-mediators of cancer cell motility and found a highly significant association with the expression of stathmin protein in BC patients, which was further supported by a clinical correlation of the expression of both genes. Stathmin is highly expressed in BC and correlates with the more aggressive forms of disease and with a bad prognosis [44–46]. Moreover, stathmin loss in a mouse model of mammary tumorigenesis (MMTV-Δ16HER2 transgenic mice) decreased the incidence and increased the latency of these very aggressive breast carcinomas [27].

The correlation between HMGA1 and stathmin was further investigated in TNBC cells showing for the first time that HMGA1 modulates stathmin expression and its activity on microtubules. In fact, the pro-tumorigenic functions of stathmin exerted by altering cytoskeleton dynamics, increasing the rate of free versus polymerized tubulin and promoting cell motility, were reversed by HMGA1 silencing.

Stathmin expression and function are regulated primarily at the post-transcriptional level, through control of protein stability and post-translational modifications, and through sequestration of stathmin by interaction with the cyclin-dependent kinase (CDK) inhibitor p27 [30]. Here, we demonstrated that depletion of HMGA1 in TNBC cells leads to increased binding of p27 with stathmin, resulting in inhibition of the depolymerization activity of stathmin on microtubules, thereby inducing decreased cell migration (Fig. 6D).

How does HMGA1 regulate p27 expression? It has been recently reported, in a mouse model of diabetic cardiomyopathy, that HMGA1 activates the transcription of miR-222, which targets p27 mRNA [47], as shown in other cellular models [48–54]. In addition, miR-222 has also been shown to target phosphatase 2 A subunit B (PPP2R2A) resulting in AKT activation [55, 56]. Activated AKT can then phosphorylate p27, leading to its cytoplasmic localization and degradation. These mechanisms might represent therefore possible ways of regulation of p27 by HMGA1 in our model as well.

Overexpression of stathmin has been shown to have a role in drug resistance. High levels of stathmin expression are associated with chemoresistance while its downregulation confers sensitivity to different drugs [37, 57–59]. Stathmin has been shown to confer resistance to paclitaxel, vinblastine, and cisplatin [35, 57, 60–62]; in particular, the resistance to paclitaxel is led by a stathmin-mediated decrease of paclitaxel binding to microtubules, thus decreasing the activity of the drug [38]. On the other side, stathmin silencing in different cancer cells, including breast, led to an increase in paclitaxel and vinblastine sensitization [37, 59–66]. Intriguingly, FOXM1, a transcription factor whose stability and transcriptional activity are increased by HMGA1 in TNBC cells [67] confers chemoresistance to paclitaxel and trastuzumab through the upregulation of stathmin in BC cells [57]. This suggests a circuit in which HMGA1 can increase stathmin activity through different, but concurrent, mechanisms. In TNBC, the mainstay of treatment is chemotherapy. Paclitaxel is frequently used in this first-line treatment of BC but, unfortunately, the appearance of chemoresistance is a great obstacle to clinical applications [68]. Our findings reported that depletion of HMGA1 in a xenograft mouse model sensitizes BC cells to paclitaxel, which would indicate that taxol-based applications may be more effective in reducing the tumour burden when tumour cells express low levels of HMGA1 (Fig. 6D). Our in vitro data as well as data from the literature [35–38] support the possibility that stathmin may mediate the effects of HMGA1 on microtubules stability and response to taxol. A definitive answer would be provided by rescue experiments in mice xenografted with cells knocked out for HMGA1 and overexpressing stathmin. It would be worth in the future to better evaluate this point and possibly investigate HMGA1 expression in BC and, also, to undertake efforts in finding drugs targeting HMGA1 that could be used in combination with taxol.

## Materials and methods

### Cell culture, transfection, and treatments

Human breast cancer MDA-MB-231, MDA-MB-157, and MDA-MB-468 cell lines were routinely grown in high glucose DMEM, with 10% tetracycline-free FBS (Euroclone, catalog # ECS0182L), 2 mM L-Glutamine (Euroclone, catalog # ECB3000D), 100 U/ml Penicillin, and 100 µg/ml Streptomycin (Euroclone, catalog # ECB3001D). Cell lines were kindly provided by the laboratory of prof. G. Del Sal (Dept. Life Sciences, University of Trieste, Italy) and tested for Mycoplasma weekly. For the stable inducible silencing of HMGA1, MDA-MB-231 TetR shCTRL (sequence: 5’-ACAGUCGCGUUUGCGACUG-3’) or shHMGA1 (sequence: 5’-GACAAGGCUAACAUCCCAC-3’) clones were kept in selection with 1 mg/ml of G418 and 5 µg/ml blasticidin (Sigma) and treated with 1 µg/ml doxycycline to induce the shRNA [19]. For siRNA silencing, Lipofectamine^TM^ RNAiMAX reagent (Invitrogen/Thermo Fisher Scientific catalogue # 13778075) was used to transfect 30 pmol of siRNA/35 mm dish. siCTRL (sequence: 5’-ACAGUCGCGUUUGCGACUG-3’), siHMGA1 (sequence: 5’-ACUGGAGAAGGAGGAAGAG-3’) and siHMGA1_1 (sequence: 5’- GACAAGGCUAACAUCCCAC-3’) have been previously used [19] (siCTRL and siHMGA1_1 have the same sequence of the shRNA TetR inducible ones used in this work). siSTMN against stathmin (sequence: 5’-CGUUUGCGAGAGAAGGAUA-3’) was designed using an Invitrogen tool. p27 was silenced with TriFECTa® RNAi Kit (IDT, catalog # hs.Ri.CDKN1B.13). Plasmid transfection was carried out using Lipofectamine 3000 (Invitrogen, catalog # L3000008), transfecting 1–2.5 µg of plasmid/35 mm dish. Vectors used were: dsRED (pDsRed-Monomer-C1, Clontech) and dsRED-STMN, in which the ORF sequence of stathmin is cloned at *Bam*HI restriction site in the dsRED vector. For the cycloheximide (CHX, Sigma, catalog # C7698) treatment, 48 h after siRNA transfection MDA-MB-231 cells were treated with 50 µM CHX and harvested at 1.5, 6, and 9 h.

### Immunoblotting and immunoprecipitation

Cells were washed in ice-cold PBS and then lysed in SDS sample buffer (62.5 mM Tris pH 6.8; 2% SDS; 10% glycerol) supplemented with protease inhibitors. Total lysates were separated by SDS-PAGE and the proteins were transferred to nitrocellulose membrane ∅ 0.2 µm (GE Healthcare, WhatmanTM) using a wet transfer system. Western blot analyses were performed according to standard procedures and using the following primary antibodies: α-HMGA1 [19], α-p27 (BD-TL, catalog # 610241), α-STMN (Cell Signalling, catalog # D1Y5A, Santa Cruz catalog # sc-55531), α-H3 (Abcam), α-β-Tubulin (Sigma, catalog # T5168), α-β-actin (Sigma, catalog # A2066), α-Vinculin (Santa Cruz, catalog # sc-25336). Immunoprecipitation experiments were performed incubating 0.7–1 mg of total MDA-MB-231 cell extract lysed in RIPA buffer (50 mM Tris HCl pH 8, 2 mM EDTA, 10 mM NaCl, 10% glycerol, 1% Triton X-100, 0.1% SDS), with the specific primary antibody in HNTG buffer (20 mM HEPES, 150 mM NaCl, 10% glycerol, 0.1% Triton X-100), overnight at 4 °C. Then, the lysate-antibody formulation was incubated with protein A or protein G Sepharose 4 Fast Flow (Amersham Biosciences, catalog # GEH17-5280-01 and GEH17-0618-01) for 1.5 h at 4 °C. After several washes in HNTG buffer, proteins were eluted from the resin by 3× Laemmli sample buffer with 50 mM dithiothreitol. Immunoprecipitations were analyzed by western blot with indicated antibodies.

### Proximity ligation assay (PLA)

MDA-MB-231 cells were seeded on coverslips and transiently silenced with siCTRL and siHMGA1, as described before. After 72 h of silencing, cells were fixed with 4% PFA. Cells were then permeabilized with 0.2% Triton/PBS and assayed for p27 and stathmin proximity, using the Duolink PLA Fluorescence kit (Millipore, Sigma, catalog # DUO92002-100RXN/DUO92004-100RXN/DUO92014-100RXN/DUO82049-4L) and manufacturer-recommended protocol. Primary antibodies used are: α-Stathmin (Cell Signaling, catalog # D1Y5A) and α-p27 (BD Transduction Laboratories, catalog # 556049). Nuclei were counterstained using TO-PRO-3 iodide (642/661) (Invitrogen, catalog # T3605). Images were acquired using TCS-SP8 Confocal Systems (Leica Microsystems) interfaced with the Leica Application Suite (LAS) software. As internal negative control, all the protocol was performed using antibody diluent alone without adding primary antibodies. Collected images were analyzed using ImageJ software.

### Cytoplasmic and nuclear protein fractionation

Cytoplasmic/nuclear fractionation was performed as previously described [69]. Briefly, MDA-MB-231 and MDA-MB-157 cells were washed in ice-cold PBS and resuspended in hypotonic buffer (10 mM Tris pH 7.4, 10 mM NaCl, 3 mM MgCl2). Swelled cell pellet was resuspended firstly in mild detergent solution (10 mM Tris, pH 7.4, 10 mM NaCl, 3 mM MgCl2, 10% glycerol, 0.5% Nonidet P-40, 0.5 mM dithiothreitol (DTT) (Sigma)) to recover cytoplasm soluble fraction, then in the mild detergent solution added with 3.3% (wt/vol) sodium deoxycholate and 6.6% (vol/vol) Tween 40 to recover cytoplasm insoluble fraction and to isolate nuclei. The two cytoplasmic fractions were put together. Laemmli sample buffer was then added to the cytoplasmic and nuclear fractions before SDS gel electrophoresis and immunoblotting with the indicated antibodies.

### Microtubule-free tubulin separation

MDA-MB-231 cells at sub-confluence were washed once with PBS at 37 °C and then lysed with Microtubule Stabilizing Buffer (20 mM Tris/HCl pH 6.8, NaCl 0.14 M, 2 M glycerol, 1 mM MgCl2, 2 mM EGTA, 0.5% Triton X-100, cOmplete Protease Inhibitor Cocktail (Roche, catalog # 11836145001), 4 µM Paclitaxel (Sigma, catalog # T7402). Cells were then centrifuged at 12,000 × *g*, 4 °C. The supernatant was collected as a free-tubulin fraction, while the pellet included the polymerized microtubules. Both fractions were subjected to western blot analysis. To normalize the amount of tubulin in different biological samples, total tubulin has been used and calculated as the sum of microtubule and free-tubulin band intensities.

### Cell migration assays

For trans-well cell migration assays, 24-well PET inserts were used (8.0 μm ∅, Corning, catalog # CC3464) and 40,000 MDA-MB-231 cells were seeded on the top. After 18–24 h, migrated cells were fixed in PFA 4% and stained with Crystal Violet 0.5% (Sigma). At least six images for insert were captured by OLYMPUS CK2 inverted optical microscope at ×10 magnification through the digital camera Canon PowerShot A630. Cells were counted with ImageJ software. Before trans-well assays, cells were differently treated. In nocodazole treatment, cells previously silenced with siRNA for 72 h, were treated with 1 µM nocodazole for 1 h before seeding in the transwells. To co-deplete HMGA1 and stathmin, cells were co-transfected at the same time and seeded in the transwells after 72 h from silencing. For wound-healing assay, MDA-MB-231 and MDA-MB-157 cells were seeded in antibiotics-free DMEM at a density of 2–3 × 10^5^ cells/well in 35 mm dish, in biological triplicates. In stathmin overexpression experiments, cells were first silenced for HMGA1 and, after 24 h, transfected with 1–2.5 µg of dsRED or dsRED-STMN for 48 h. In experiments where we co-depleted HMGA1, p27 and stathmin, cells were firstly silenced for p27. Then, after 24 h, siHMGA1 and siSTMN were applied for 48 h. Cells were then scraped with a 200 μl tip, and wound closure has been followed for 4 or 7 h depending on the cell line used. Images were taken for each well and the wound areas were analyzed by ImageJ software.

### Immunostaining

For stathmin and tubulin analysis on migrating cells, MDA-MB-231 and MDA-MB-157 cells were seeded in antibiotics-free DMEM on glass coverslips at a density of 4–6 × 10^4^ cells/well in a 24-multiwell plate in biological triplicates. Cells were then silenced for HMGA1 and, for MDA-MB-157 cells, after 24 h, transfected with 1–2.5 µg of dsRED or dsRED-STMN for 48 h. After that, cells were scraped with a 200 µl tip and fixed after 4 h with PFA 4%. For p27 localization analysis MDA-MB-231 and MDA-MB-157 cells were grown on glass coverslips and silenced for HMGA1 for 72 h and then fixed in 4% PFA.

After fixation, cells were permeabilized in a solution with 0.3% of Triton X-100/PBS and saturated in 5% BSA/PBS. Subsequently, cells were incubated with the following primary antibodies: α-p27 (N-20) (Santa Cruz, catalog # sc-527), α-STMN (Cell Signalling, catalog # D1Y5A), α β-Tubulin (Sigma, catalog # T5168). As secondary antibodies we used α-rabbit IgG Alexa Fluor^®^ 647 (Thermo Fisher Scientific, catalog # A31573), α-mouse IgG Alexa Fluor^®^ 488 (catalog # A32723), and α-rabbit IgG Alexa Fluor^®^ 488 (catalog # A32731). Nuclei were stained with Hoechst 33342. Images were acquired using Elyra7 lattice SIM super-resolution microscope (Zeiss) with pco.egde sCMOS camera and ZEN Black software or a Nikon Eclipse e800 microscope with Nikon ACT-1, then images were analyzed with ImageJ.

### Gene expression analysis

Total RNA was isolated following the manufacturer’s instructions for the TRIzol reagent (Invitrogen, catalog # 15596018). The isolated RNA was subjected to DNase I (Invitrogen, # 18068015) treatment and to a subsequent phenol/chloroform purification. For quantitative RT-PCR (qRT-PCR), mRNA was reverse transcribed with Random primer by the Superscript III (Invitrogen, catalog #11732088), according to the manufacturer’s instructions. The CFX96 Real-Time PCR detection system (Bio-Rad) was used to perform PCR, carried out by iQTM SYBR Green Supermix (Bio-Rad, catalog # 170-8882). The following primers were used: for GAPDH, 5’-TCTCTGCTCCTCCTGTTC-3’ (forward) and 5’- GCCCAATACGACCAAATCC-3’ (reverse); for HMGA1, 5’-ACCAGCGCCAAATGTTCATCCTCA-3’ (forward) and 5’-AGCCCCTCTTCCCCACAAAGAT-3’ (reverse); for p27, 5’-AGCAATGCGCAGGAATAAGG-3’(forward) and 5’-TTCTGAGGCCAGGCTTCTTG-3’ (reverse); for STMN, 5’-AATGGCTGCCAAACTGGAAC-3’ (forward) and 5’- TCTCGTCAGCAGGGTCTTTG-3’ (reverse). The data obtained were analyzed with Bio-Rad CFX Manager software and the relative gene expression was calculated by ΔΔCt method, using the GAPDH as a normalizer.

### In vivo experiments

Female athymic (nude) mice, 6–7 weeks old, were purchased from Charles River Laboratories. The animals were allowed to acclimate for 7 days before the study initiation. All of the animals were housed under pathogen-free conditions and were given water and chow ad libitum. Animal care and use were in accordance with Institutional and NIH guidelines. 2 × 10^6^ cells/0.1 ml MDA-MB-231 shCTRL and shHMGA1 cells were subcutaneously injected into the mammary fat pad of female athymic mice. To induce shRNA, mice were administered drinking water supplemented with 2% sucrose plus 1 mg/ml doxycycline. The treatment with 12 mg/kg of paclitaxel (Taxol®, Actavis) started when tumours reached 50–100 mm^3^. The drug was given three times a week for 15 times, the time by which mice were sacrificed. During the treatments, tumour volumes were measured three times/week with a calibre, and the tumour weights were calculated as follows: [length (mm) × width (mm)^2^]/2.

### Breast cancer datasets

To evaluate the enrichment of p27 and stathmin in BC samples the TCGA (The Cancer Genome Atlas) dataset on the Memorial Sloan Kettering Cancer Genomic Portal (http://www.cbioportal.org/public-portal) was investigated. We also used Memorial Sloan Kettering Cancer Genomic Portal to investigate the correlation between the RNA expression of HMGA1 and p27 or HMGA1 and STMN in 844 breast tumours contained in the TCGA Invasive Breast Carcinoma dataset [70]. The correlation was evaluated by the Pearson rank correlation coefficient test. To evaluate the correspondence between the p27 and stathmin expression levels among BC clinical data, we employed the Gene expression-based Outcome for Breast cancer Online (GOBO) web tool. To perform survival analysis, we used the Kaplan–Meyer plotter (https://kmplot.com/analysis/index.php?p=background) with Relapse Free Survival (RFS) on a cohort of 3951 BC patients and Distant Metastasis Free Survival (DMFS) on a cohort of 1746 BC patients, as a read-out.

### Statistical analysis

Data were analyzed by a two-tailed Student’s *t*-test and two-way ANOVA test, and results were considered significant at a *p*-value < 0.05. Specifically, a *p*-value < 0.05 is indicated with *, a *p*-value < 0.01 with **, and a *p*-value < 0.001 with ***. The results are presented as the mean and standard deviation (±SD) or standard error of the mean (±SEM).

## Supplementary information

Supplementary figures

Supplementary information

Original western blots

Supplementary Table 1

Supplementary Table 2

Reproducibility checklist

## Data Availability

All data supporting the findings of this study are available from the corresponding authors on reasonable request.
