# Supplementary material for: HMGA1 positively regulates the microtubule-destabilizing protein stathmin promoting motility in TNBC cells and decreasing tumour sensitivity to paclitaxel
Source: Cell Death Dis. 2022 May 3;13(5):429. doi: 10.1038/s41419-022-04843-4 (PMC9065117; doi:10.1038/s41419-022-04843-4)

Supplementary Figures

Supplementary Fig.1

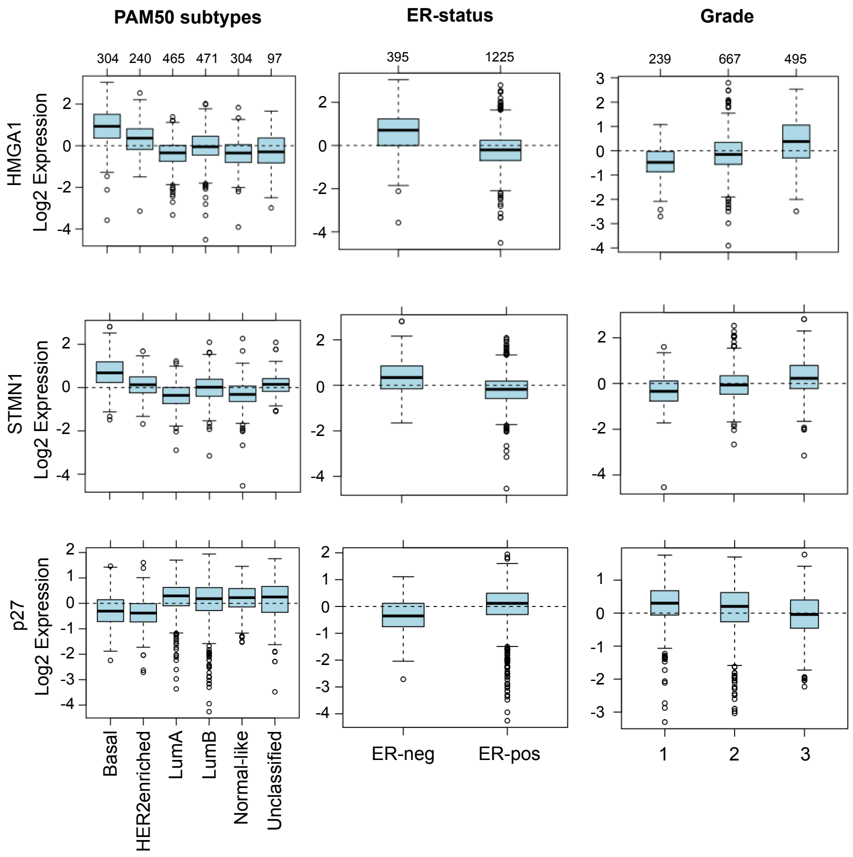

Supplementary Fig.2

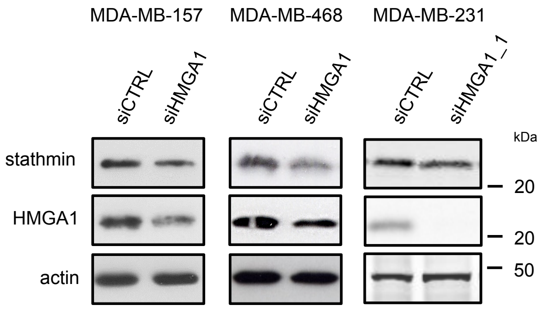

Supplementary Fig.3

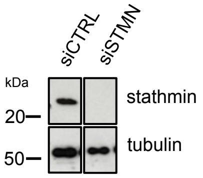

Supplementary Fig.4

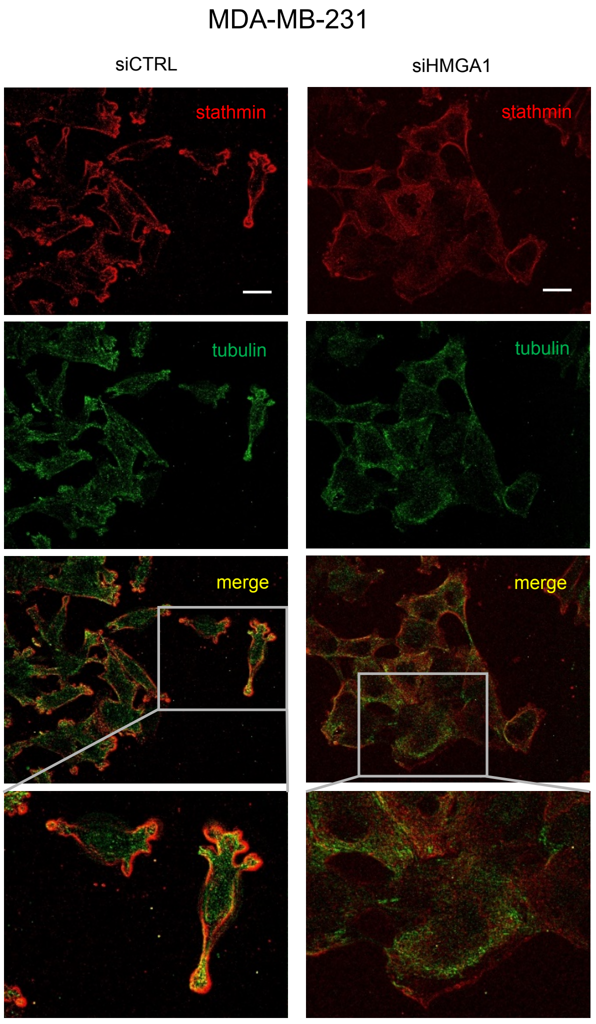

Supplementary Fig.5

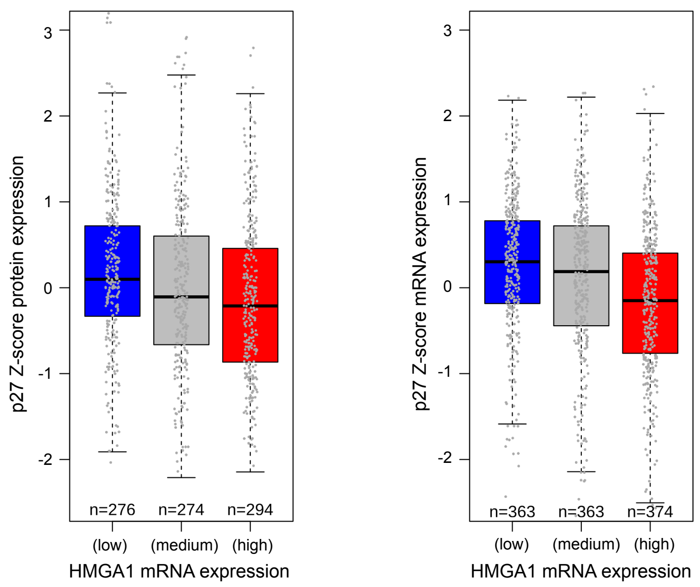

Supplementary Fig. 6

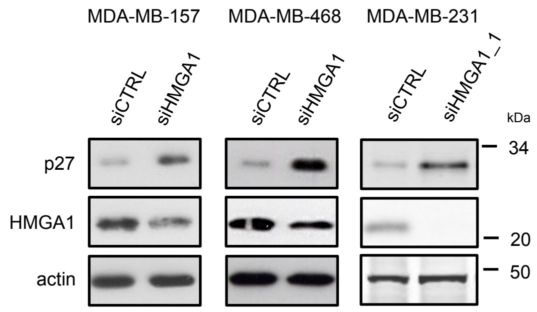

Supplementary Fig.7

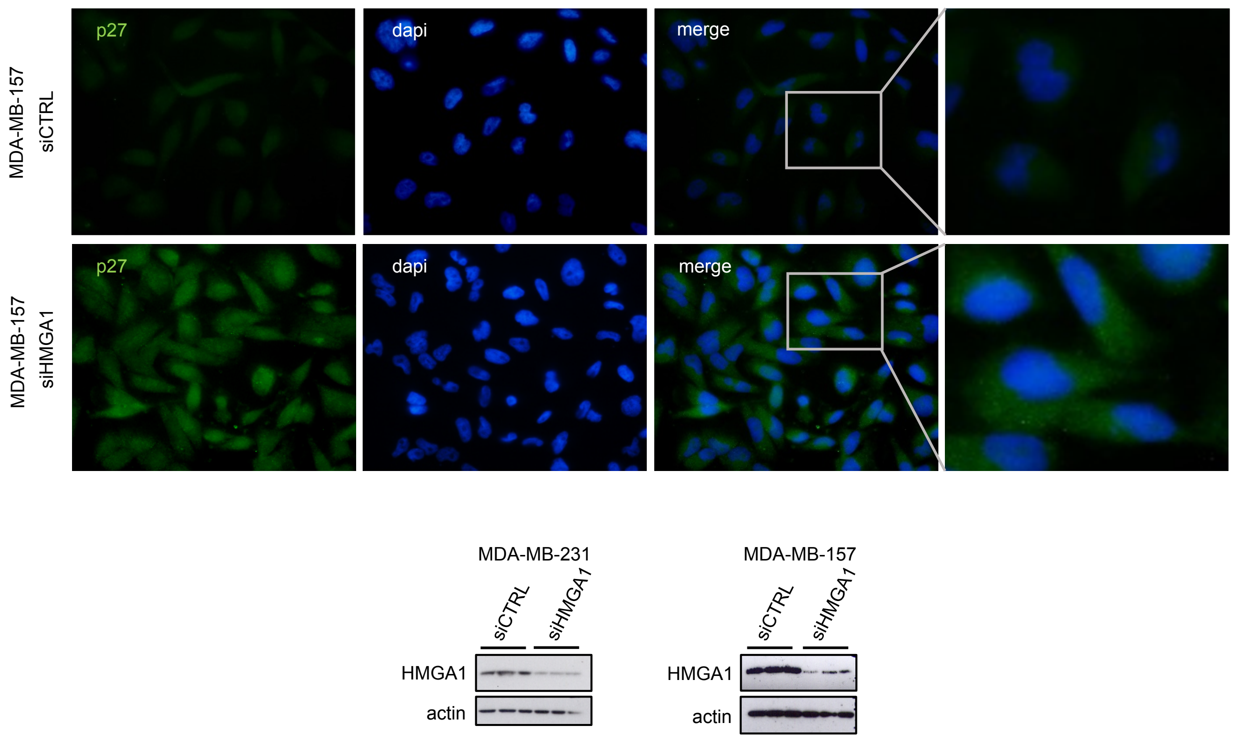

Supplementary Fig. 8

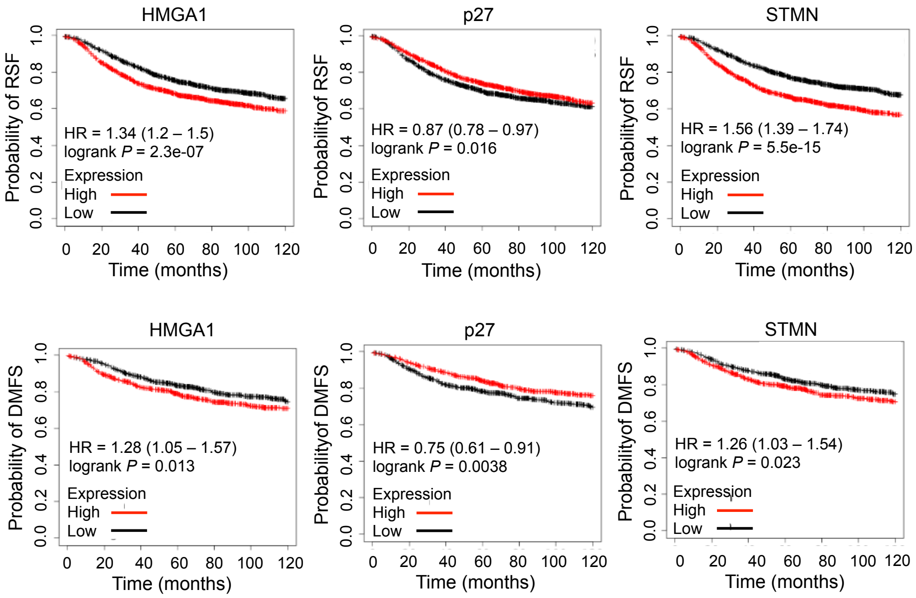

Supplement: Supplementary file 1 — Supplementary figures [file 41419_2022_4843_MOESM1_ESM.pdf]
