# Supplementary material for: HMGA1 positively regulates the microtubule-destabilizing protein stathmin promoting motility in TNBC cells and decreasing tumour sensitivity to paclitaxel
Source: Cell Death Dis. 2022 May 3;13(5):429. doi: 10.1038/s41419-022-04843-4 (PMC9065117; doi:10.1038/s41419-022-04843-4)
Supplement: Supplementary file 2 — Supplementary information [file 41419_2022_4843_MOESM2_ESM.docx]

**SUPPLEMENTARY FIGURES**

**Supplementary Fig. 1:** Expression of HMGA1, STMN and p27 among BC subtypes, ER-status, and tumour grade. The analyses were performed investigating the Gene expression-based Outcome for Breast cancer Online (GOBO) tool.

**Supplementary Fig. 2:** Representative western blot analysis of stathmin in MDA-MB-157 and MDA-MB-468 cells 72h after HMGA1 silencing with siHMGA1 and in MDA-MB-231 cells with siHMGA1_1. Actin is used as a loading control (n = 3).

**Supplementary Fig. 3:** Control Western blot analyses of total stathmin and tubulin levels in MDA-MB-231 cells silenced or not for stathmin expression.

**Supplementary Fig. 4:** Representative immunofluorescence analysis of stathmin (red) and tubulin (green) at the migratory front of MDA-MB-231 cells silenced or not for HMGA1. After 72h from silencing, a scratch was performed on the cell culture. Cells were allowed to migrate for 4h and then fixed and stained with specific antibodies. ImageJ was used to focus the staining on the edges of the cells. For each condition a further magnification of the picture is reported below the merged staining. Images were taken at 60X magnification. Scale bar, 20 µm.

**Supplementary Fig. 5:** Boxplot analysis showing the correlation between the mRNA expression of HMGA1 with p27 protein (left) and mRNA (right) expression in a cohort of 844 TCGA breast cancer patients. In blue are highlighted the tumour samples of patients with low HMGA1 expression levels, in grey the tumour samples with medium HMGA1 expression levels and in red the tumour samples with high HMGA1 expression levels.

**Supplementary Fig. 6:** Representative western blot analysis of p27 in MDA-MB-157 and MDA-MB-468 cells 72h after HMGA1 silencing with siHMGA1 and in MDA-MB-231 cells with siHMGA1_1. Actin is used as a loading control (n = 3). Panels showing HMGA1 and actin are the same as in supplementary Figure 2 because they belong to the same experiment.

**Supplementary Fig. 7:** Representative immunofluorescence analysis of p27 localization in MDA-MB-157 cells after 72h of HMGA1 silencing. On the right, magnification of the merged staining. Images were taken at 60X magnification. Below, HMGA1 silencing in MDA-MB-231 and MDA-MB-157 cells used for nucleus/cytoplasm fractionation.

**Supplementary Fig. 8:** Kaplan-Meier survival curves of Relapse Free Survival (RFS) of 3951 breast cancer patients and Distant Metastasis Free Survival (DMFS) of 1746 breast cancer patients investigated with Kaplan-Meier plotter (KM-plotter, https://kmplot.com/analysis/index.php?p=background) and containing information from GEO, EGA and TCGA. The data investigated were based on the RNA expression of HMGA1, p27 and STMN. Red line: number of patients with high levels of specified gene expression; Black line: number of patients with low levels of gene expression.
