# Supplementary material for: HMGA1 positively regulates the microtubule-destabilizing protein stathmin promoting motility in TNBC cells and decreasing tumour sensitivity to paclitaxel
Source: Cell Death Dis. 2022 May 3;13(5):429. doi: 10.1038/s41419-022-04843-4 (PMC9065117; doi:10.1038/s41419-022-04843-4)

Fig.1 Uncropped Western Blots

E

MDA-MB-231

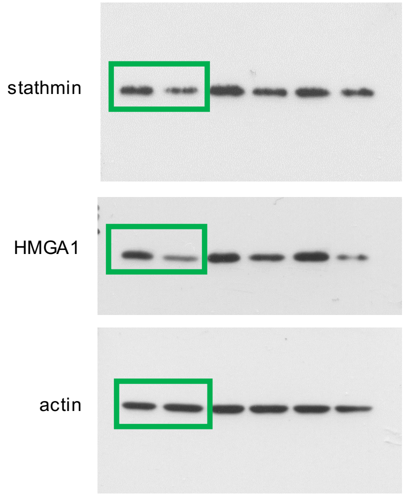

Fig.2 Uncropped Western Blots

A

MDA-MB-231

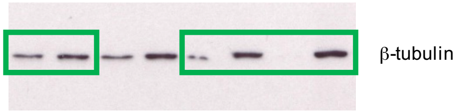

B

MDA-MB-231

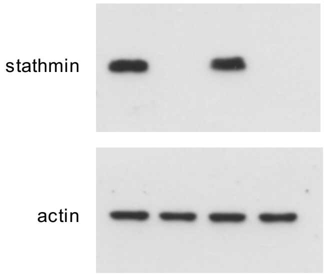

Fig.3 Uncropped Western Blots

A

MDA-MB-231

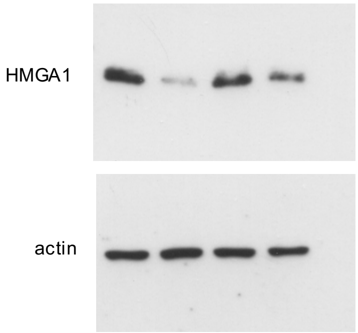

B

MDA-MB-231

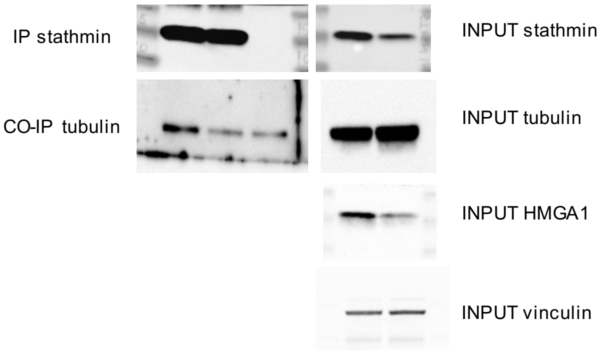

C

MDA-MB-231

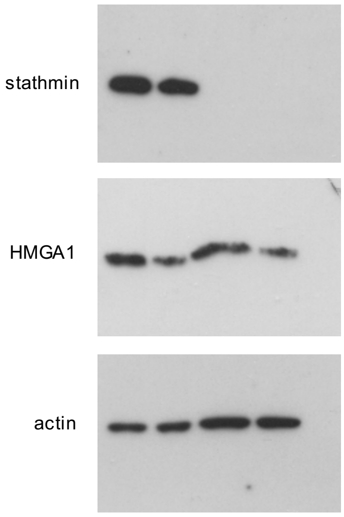

D

MDA-MB-231

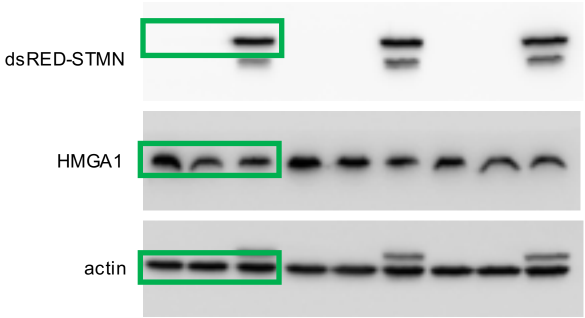

MDA-MB-157

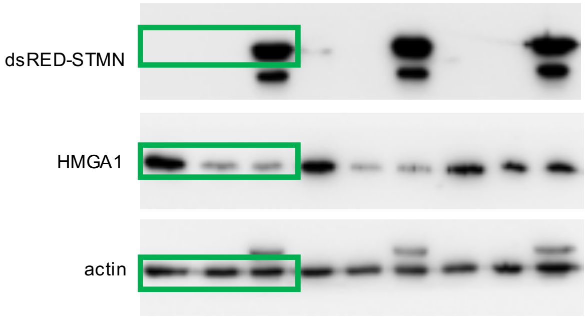

Fig.4 Uncropped Western Blots

A

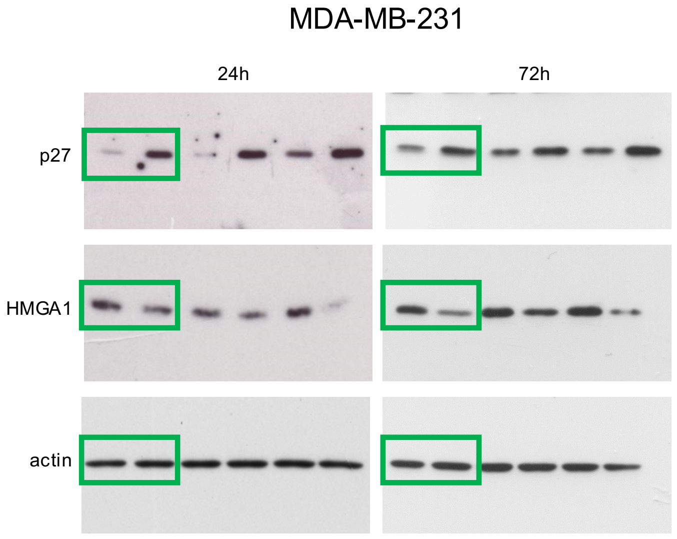

B

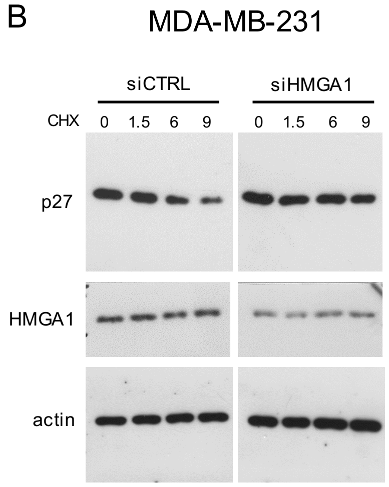

C

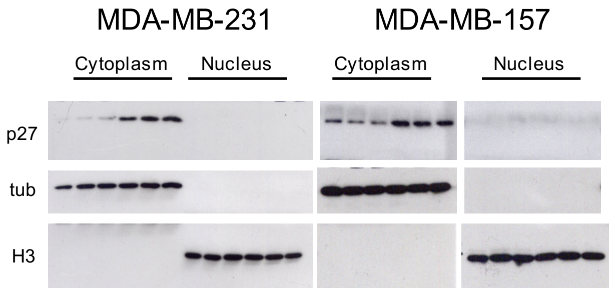

Fig.5 Uncropped Western Blots

A

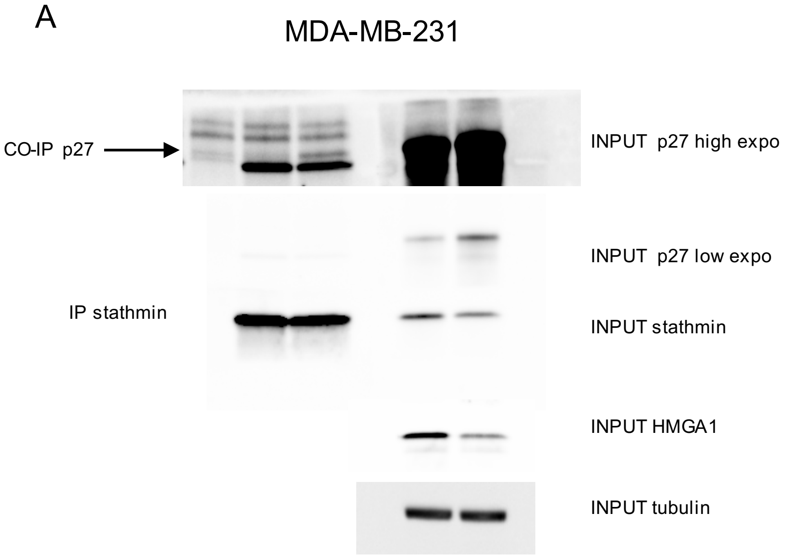

C

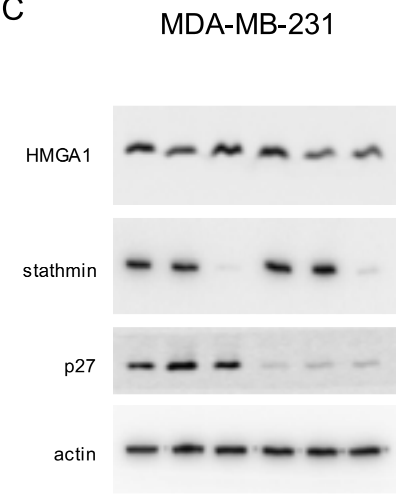

Supplement: Supplementary file 3 — Original western blots [file 41419_2022_4843_MOESM3_ESM.pdf]
